# COVID-19 pandemic: Is it the right time to develop interconnected national biomedical registries?

**DOI:** 10.5808/gi.21021

**Published:** 2021-12-31

**Authors:** Athanasios S. Kotoulas

**Affiliations:** Department of Food Science & Nutrition, University of Thessaly, Karditsa 43100, Greece

Biomedical data storage procedures and reuse availabilities are essential for high-quality healthcare, therapeutic protocols improvements, pharmacy vigilance, and public health surveillance. Healthcare personnel and information have recognized the continuous need for the improvement of electronic health records and interoperability among different healthcare/hospital information systems [1]. Moreover, prior research has indicated that the integration of molecular/genomic and clinical data is unquestionably necessary and valuable to fight against the majority of diseases. In biomedical research, relative literature accompanied by clinical and molecular data repositories as well as coordinated actions have already been implemented worldwide to enhance international bio-data exchange and cross-country comparison of healthcare performance [2,3]. Although national-scale electronic registries, healthcare initiatives, and data sharing approaches have been emerged to promote individual medicine, there is still much international effort to accomplish bio-data sharing and overcome sensitive patient data issues.

On the one hand, the coronavirus disease 2019 (COVID-19) outbreak has already influenced several human social activities and has changed the modern standard of life. Throughout history, humankind has faced and recorded the consequences of prior epidemics by finding solutions and adopting epidemic response policies [4,5]. Most healthcare systems and medical staff are overstretched every day worldwide. Furthermore, the selective lockdown remains the last of a series of restrictive government measures enacted to fight the spread of the virus and maintain the daily operation of health units at manageable levels as much as possible [6,7]. On the other hand, clinical studies/trials are always at the top of scientific biomedical efforts and research. Since scientists became aware of the discernible risk of new severe acute respiratory syndrome coronavirus (SARS-CoV) strains, more than 4,000 clinical studies/trials have been submitted to the Clinical Trial. Gov database. The recent announcements regarding the SARS-CoV-2 vaccine solutions and the various pharmaceutical interventions have brought hope to the world from a therapeutic and an economic standpoint [8,9]. Additionally, several digital transformation paradigms have emerged through the pandemic, globally. From a health IT perspective, the healthcare crisis has triggered computer scientists and IT companies to develop high-speed networks and software to compensate for travel restrictions and the closure of businesses and educational structures [10,11].

In 2019, we proposed a national biomedical registry framework for the enhancement of clinical research using free open-source software (FOSS). We focused on a data entry framework for the genomic and molecular information of a patient [12]. In addition, in translating multiple query results from the ClinicalTrials.gov database with mesh terms [[(‘any condition)’ AND [(‘mutation’) OR (‘SNP’) OR (‘gene’) OR (‘genetic’) OR (‘allele’)]] to a proposed eGov health policy (Fig. 1), we developed the following generic proposals: (1) “*If every country develops a web-based national biomedical registry, the genomic/molecular information of a patient should be a priority in its design”, and (2) “Biomedical Informatics will play a vital role in the implementation of a national biomedical registry in every country, worldwide*”. Moreover, when we searched the ClinicalTrials.gov database, we found more than 100 genomic-related COVID-19 clinical studies/trials. After reviewing the modern biomedical research, our team believes that a personal bio-molecular signature should be registered either via a custom national healthcare application or via the generic modular parts of the structured electronic healthcare record of a hospital information system. Academic and commercial streamline software including bio-molecular database structures (i.e., DNA mutations, SNPs, PCR results) and further technical details (i.e., Flat files, VCF files, binary large objects, usage of molecular biology/bioinformatics databases annotations) have been published over a decade to integrate cross-country clinical and molecular databases [13-17]. Recent advanced technology, combining NoSQL databases, molecular/genomic standard data structures, cloud architectures, reliable FOSS, and highs performance computing servers, seems promising to efficiently manage large next-generation sequencing/whole genome sequencing data and distribute via web services individuals’ bio-information [18-20].

Therefore, it is highly possible that IT management and eGov policies can create positive change in the pandemic. If the clinical researchers could rapidly find eligible patients or a cohort of healthy individuals for clinical research, based on their biomedical information and via interconnected web frameworks, then the personalized medicine and vaccine development research would be enhanced. The global healthcare IT community should focus on the implementation of a generic structured biomedical registry in every country. Without a doubt, a centralized national biomedical patient record could contribute to both solving long-term problems and research on rare diseases, as well as epidemiological studies. In the present infectious disease era, all countries must support biomedical cooperation and the development of interoperable health IT systems in parallel to other crucial data collection viewpoints. Maintaining the security of a person’s sensitive information, electronic biomedical data interchange that is used to suppress a pandemic could be supported through the development of web-based national biomedical registries [20,21]. Regardless of the COVID-19 outbreak, the next generation of integrated hospital information systems will play a key role in healthcare innovation globally [22-25]. A possible obstacle in this direction is the extra burden that may fall on healthcare staff. Especially the medical staff is burdened with the task of entering and updating patient health data. It is also a fact that medical staff needs to improve their health informatics skills to be able to efficiently exploit healthcare software [20,24,25]. Consequently, health information exchange, health IT skills, modern web services solutions, and interoperability between hospital information systems and molecular diagnostics laboratories, are parameters of the same equation for common healthcare policies.

As a registered nurse, molecular biologist, and informatician scientist, I strongly believe that healthcare personnel are destined to play a key aspect in the proposed interconnected national biomedical registries. Furthermore, clinical and laboratory processes within a hospital should be applied to electronic health records and other healthcare software documentations. In the generic digital transformation that has already come from the COVID-19 pandemic, healthcare personnel could further assist in storing the individual, clinical, and genomic profile of each patient. In this way, the objective purposes of the proposed biomedical registries, the clinical research, and the levels of biomedical knowledge and research will be significantly promoted. Thinking to the future, I am convinced that Biomedical & Genomics Informatics will be at the forefront of the desired qualifications of health professionals, and every designed step for a new Public Health Policy by any government healthcare administration will be followed by the solutions derived from Health Informatics.

## Figures and Tables

**Fig. 1.**
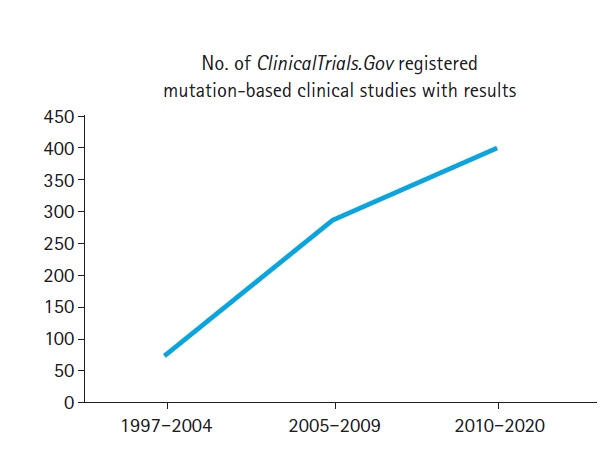
ClinicalTrials.Gov database example query [‘any condition’ AND ‘mutation’ AND ‘completed with results’]: number of registered mutation-based clinical studies with results.
